# An upconversion luminescence and temperature sensor based on Yb^3+^/Er^3+^ co-doped GdSr_2_AlO_5_†

**DOI:** 10.1039/c7ra13759a

**Published:** 2018-03-05

**Authors:** Gangyi Zhang, Qinping Qiang, Shanshan Du, Yuhua Wang

**Affiliations:** Department of Materials Science, School of Physical Science and Technology, Lanzhou University Lanzhou 730000 PR China wyh@lzu.edu.cn +86 931 8913554 +86 931 8912772; Key Laboratory for Special Function Materials and Structural Design of the Ministry of Education, Lanzhou University Lanzhou 730000 China

## Abstract

GdSr_2_AlO_5_:Yb^3+^/Er^3+^ micro-particles were synthesized by a simple solid state method. The structure, morphology, size and upconversion luminescence features have been characterized. These results indicated that GdSr_2_AlO_5_ has a contracted tetragonal cell and has irregular block shaped particles with sizes of about 5 μm. During upconversion, green (^2^H_11/2_, ^4^S_3/2_ → ^4^I_15/2_) (527 nm, 549 nm) and red (^4^F_9/2_ → ^4^I_15/2_) (665 nm) emissions had been observed, both of which occurred *via* a two-photon population process. In addition, green UC emission characteristics were studied, and it was found that its temperature ranged from 293 K to 473 K and the sensitivity was 0.0054 K^−1^ at 473 K. This indicated that GdSr_2_AlO_5_:Yb^3+^/Er^3+^ micro-particles may have potential application in high temperature environments for safety signs.

## Abstract 2

GdSr_2_AlO_5_:Yb^3+^/Er^3+^ micro-particles were synthesized by a simple solid state method.

## Graphical abstract

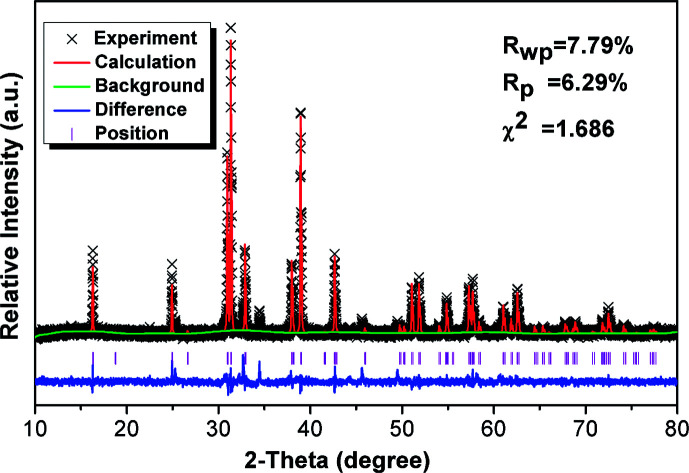

## 1. Introduction

Upconversion (UC) luminescence materials are luminescent materials that can convert a near-infrared excitation into a visible emission through lanthanide doping.^1^ These materials have attracted extensive interest in the past decade because of their unique features, such as high photochemical stability, narrow emission bandwidths, and large anti-Stokes shifts, making them useful in optical communication, lasers, sensors, biomedical analyses and so on.^2–4^ For efficient UC emission, Yb^3+^ ions are typically selected as sensitizers together with Er^3+^, Tm^3+^ and Ho^3+^ as activators doped into various kinds of hosts.^5,6^ Tb^3+^ and Dy^3+^ are also activators but they not often been used.^7,8^ Yb^3+^ has a larger absorption cross-section than those of rare earth activators when excited by 980 nm laser. What is more, the ^2^F_7/2_ → ^2^F_5/2_ transition of Yb^3+^ is exactly resonant with many f–f transitions of Er^3+^, Ho^3+^ and Tm^3+^ which cause to the efficient energy transfer from Yb^3+^ ions to these ions. Thence, Yb^3+^ is used extensively as sensitizer as the absorption section is much larger around 980 nm wavelength and can lead efficient energy transfers to Er^3+^, Ho^3+^and Tm^3+^. Because of its beneficial electronic energy level structures. Er^3+^ ions have been widely studied and a large number of Er^3+^ doped UC materials have been reported such as NaYF_4_:Yb,Er, NaYF_4_:Nd,Yb,Er, NaGdF_4_:Yb,Er Ba_2_Y(BO_3_)_2_Cl:Yb,Er, and Y_2_O_3_:Yb,Er.^9–13^

Temperature-sensitive luminescence materials can provide a non-contact measurement to detect the temperature by probing the dependence of emission intensities on temperature.^14^ We can make the calibration by analyzing the changes on the relative emission intensities when the temperature of the samples increase. The measurement conditions and time exposure can be simple and convenient by this fluorescence intensity ratio (FIR) technique compared with common temperature monitoring devices.^15–17^ The premise of using this method is two thermally coupled levels of RE^3+^ ions. Fortunately the Er^3+^ ions have two thermally coupled energy levels, ^2^H_11/2_ and ^4^S_3/2_, with a small energy gap around 770 cm^−1^ which is just conform to this technique.

Host materials play an important role for the UC luminescence. For purpose of obtaining efficient UC emission, lower phonon energies host materials are required because the value of nonradiative decay rates in these hosts would be minimum.^18,19^ Thence, rare earth doped fluoride crystals have been studied for a long time due to their low phonon thresholds. However, it is known to all that physical properties of fluoride crystals could not compared with oxide hosts such as chemical stability and mechanical strength. For this reason, it is still important to find new oxide host materials with efficient UC luminescence. Recently, some literatures on the UC luminescence of RE-doped oxide crystals such as Y_2_O_2_S, SrWO_4_, CaWO_4_ and Zn_3_Ga_2_GeO_8_ have been reported.^13,20–22^ Many compounds with Gd ions have been reported due to its excellent magnetic properties. And it is possible to use these compounds as multifunctional materials in various fields. There is little difference in radius of the Yb and Er ions compared with the Gd ions. So rare earth ions as luminescent centers are more easily incorporated into the host, resulting in effective emission. Moreover, aluminate phosphors have been studied extensively by virtue of their cheap raw materials, good chemical and physical stability. In a word, the Yb and Er ions can easily doped in the GdSr_2_AlO_5_ host and the as prepared GdSr_2_AlO_5_ samples are stable enough to work in high temperature and the good magnetic properties would be significant and meaningful for the potential application in biomedical imaging. The structural and optical properties of the Ce^3+^ doped solid solutions GdSr_2_AlO_5_ and Sr_3_AlO_4_F are reported by Won Bin Im *et al.*^23^ Lately, yellow emitting phosphor GdSr_2_AlO_5_:Ce^3+^^24^ and afterglow phosphors GdSr_2_AlO_5_:RE^3+^ (RE^3+^ = Eu^3+^,Sm^3+^, Pr^3+^ and Dy^3+^)^25^ have been investigated as well. These facts illustrated that the GdSr_2_AlO_5_ host is a favorable optical material, but there is no report on its UC luminescence. So based on these above points, in our work, the GdSr_2_AlO_5_ was studied as a new UC host.

In this paper, Yb^3+^/Er^3+^ co-doped GdSr_2_AlO_5_ were prepared, Yb^3+^ were added as the sensitizers with Er^3+^ as the activators due to the efficient energy transfer from Yb^3+^ to Er^3+^. Bright UC emissions from the samples can been seen under 980 nm diode laser excitation. Additionally, the possible mechanism of Yb^3+^ sensitization to Er^3+^ on tuning of the UC emission properties were discussed and concluded. The thermometry properties based on the green UC emissions of Er^3+^ were investigated in detail as well.

## 2. Experimental

### 2.1. Synthesis

GdSr_2_AlO_5_:RE^3+^ (RE^3+^ = Yb^3+^ and Er^3+^) samples were synthesized by a simple high temperature solid state reaction method. Raw materials Gd_2_O_3_ (99.9%), SrCO_3_ (99.9%), Al(NO_3_)_3_·9H_2_O (99.0%) and RE oxides (Yb_2_O_3_ and Er_2_O_3_ 99.9%) were weighed out according to the stoichiometric ratio. After all the ingredients were fully mixed evenly, the mixtures were placed into a corundum crucible and then put into a SiC rods furnace to be heated to 1510 °C for 4 h. Finally, after calcination, the samples were cooled down to room temperature in the furnace and ground into powders for further use.

### 2.2. Characterization

We use a Bruker D2 PHASER X-ray diffractometer with graphite monochromator using Cu Kα radiation (*λ* = 1.54184 Å) to determine the structure purity by X-ray powder diffraction (XRD). And the test conditions are 30 kV and 15 mA with a scanning step of 0.02° in the 2*θ* range from 10° to 80°.The morphologies of the prepared samples were inspected by a thermal field emission scanning electron microscopy (FESEM, TESCAN, MIRA3 XMU). The UC luminescence spectra were measured by using a 980 nm laser diode as the excitation source, with the HORIBA Jobin Yvon Fluorlog-3 Spectro fluorometer system.

## 3. Results and discussion

### 3.1. Structure and morphology

**Fig. 1:**
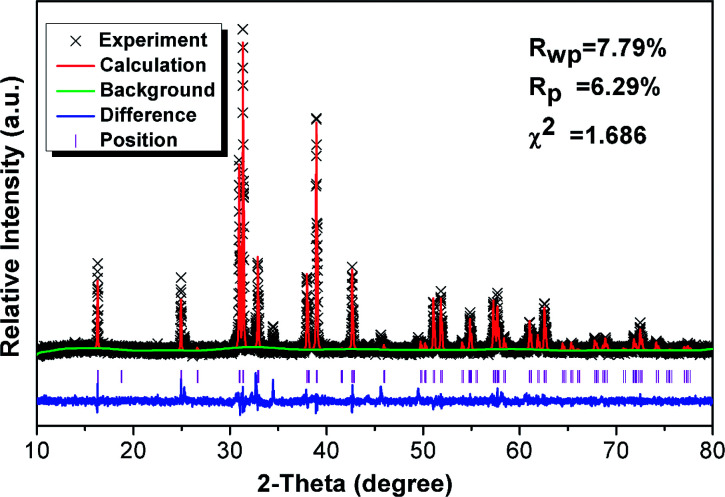
XRD refinement results for the GdSr_2_AlO_5_ host.

Fig. 1 shows the Rietveld structural refinement of the XRD pattern of the GdSr_2_AlO_5_ host obtained using the MS program based on the reported tetragonal EuSr_2_AlO_5_ in JCPDS card with no.70-2197. The black crosses, red line and green line are corresponds to the calculated pattern, experimental pattern and background, respectively. The violet short vertical lines show the positions of the Bragg reflections of the calculated pattern. The difference between the experimental and calculated patterns is plotted by the blue line at the bottom. The sample can be found to crystallize in the tetragonal space group *I* 4/*m c m* (no. 140). The refinement finally converged to *χ*^2^ = 1.686, *R*_wp_ = 7.79% and *R*_p_ = 6.29%, indicating that our prepared samples is single phase because all the observed peaks suit the reflection conditions. Furthermore, the lattice constants of GdSr_2_AlO_5_:6%Yb^3+^/1%Er^3+^ are *a* = *b* = 6.70508 (ref. 12) Å, *c* = 10.90337 (ref. 22) Å, *α* = *γ* = *β* = 90°.

**Fig. 2:**
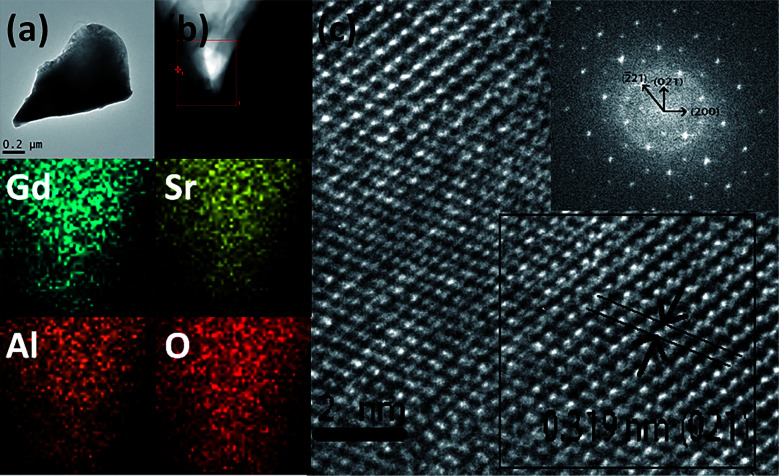
TEM (a), mapping (b), HRTEM and FFT images (c) of GdSr_2_AlO_5_ host.

Fig. 2 presents the TEM, elemental mapping, HRTEM and FFT images of GdSr_2_AlO_5_ host. The mapping images indicate a homogeneous distribution of Gd, Sr, Al and O. The HRTEM image exhibits a clear lattice fringe with lattice interplanar spacings of 3.19 Å, corresponding to the (021) plane of GdSr_2_AlO_5_. The HRTEM and FFT images demonstrate that the as prepared sample has a tetragonal crystal structure, which is the same as the XRD result.

**Fig. 3:**
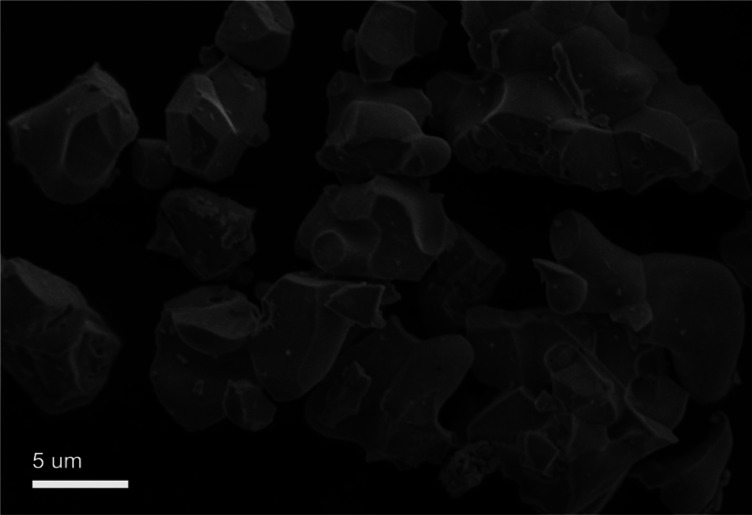
SEM image of GdSr_2_AlO_5_:6% Yb^3+^/1% Er^3+^.

As a typical sample the SEM image of the GdSr_2_AlO_5_:6%Yb^3+^/1%Er^3+^ is shown in Fig. 3. It can been find that some of these particles were reunited and the grains have irregular blocky particle shapes with sizes around 5 μm. And the SEM image of the GdSr_2_AlO_5_ host is show in Fig. S1,† which is almost the same as Fig. 3.

**Fig. 4:**
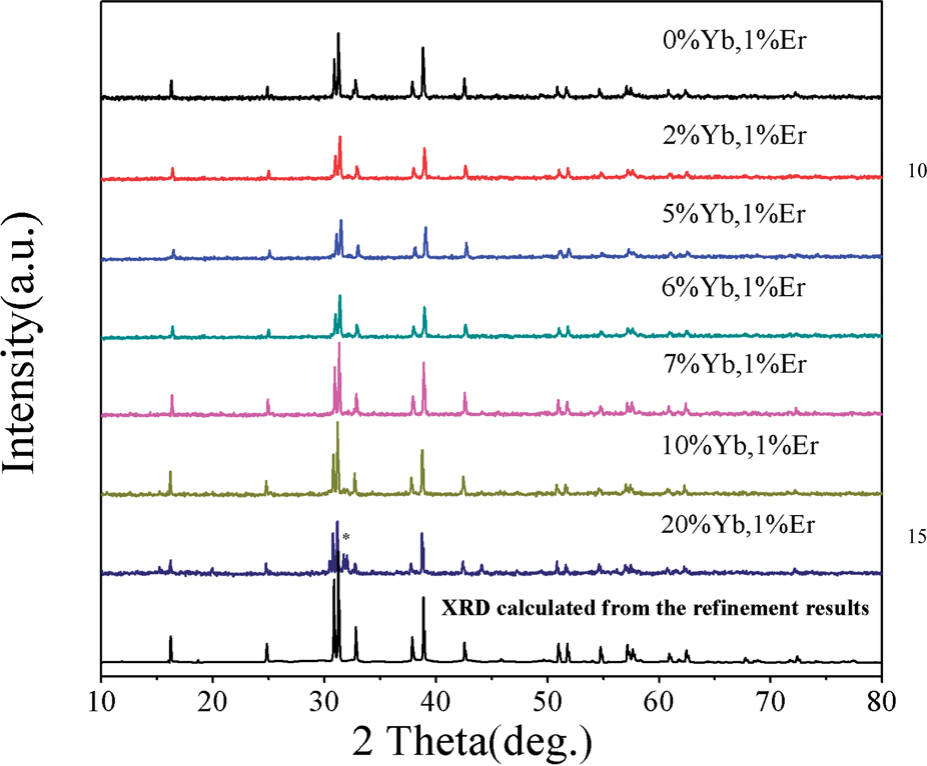
XRD patterns of GdSr_2_AlO_5_: Yb^3+^, Er^3+^.

Fig. 4 shows the XRD patterns of various RE ion-doped GdSr_2_AlO_5_ samples as well as the experimental and calculated XRD patterns of the GdSr_2_AlO_5_ host according to the refinement results. It is clear that the XRD profiles are well fitted with the calculated XRD pattern, and all these diffraction peaks of these phosphors can be accurately assigned to the GdSr_2_AlO_5_ host. It can be seen that when the Yb^3+^ content increased from 0 to 10%, the phase is still the same as GdSr_2_AlO_5_ host. So the introduction of lanthanide ions (Yb^3+^ and Er^3+^) would not change the phase structure of GdSr_2_AlO_5_ except when the Yb^3+^ doping concentration is 20%. The change of the phase structure means new phase appears with the substantial increase of Yb^3+^. This phenomenon implies the successful incorporation of Yb ions into the GdSr_2_AlO_5_ host.

Furthermore, the experimental values of Gd:Sr:Al:Yb:Er in the as-synthesized 1, 2, 3, 4, 5 and 6 samples are determined by ICP-AES (Table 1), which are all close to the theoretical values.

**Table 1:** Ions content in GdSr_2_AlO_5_ samples by ICP-AES

	Samples	1	2	3	4	5	6
Gd	Theoretical content	0.99	0.97	0.94	0.93	0.92	0.89
	Experimental	0.91167	0.90009	0.88310	0.96490	0.95768	0.93287
Sr	Theoretical content	2	2	2	2	2	2
	Experimental	2	2	2	2	2	2
Al	Theoretical content	1	1	1	1	1	1
	Experimental	1.01010	0.93194	0.95639	1.05427	1.07315	1.07779
Yb	Theoretical content	0	0.02	0.05	0.06	0.07	0.10
	Experimental	0	0.01796	0.05127	0.06522	0.07615	0.10998
Er	Theoretical content	0.01	0.01	0.01	0.01	0.01	0.01
	Experimental	0.00866	0.00947	0.01067	0.01109	0.01010	0.01001

### 3.2. UC emission properties

**Fig. 5:**
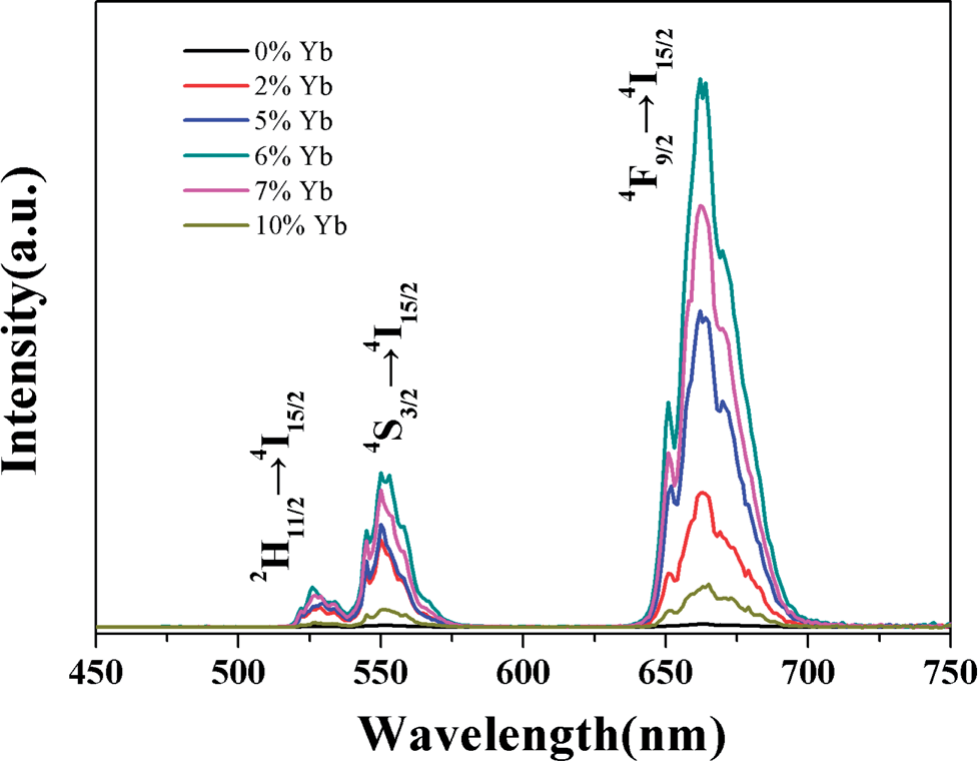
UC spectra of GdSr_2_AlO_5_:Yb^3+^,1%Er^3+^ crystals with different Yb^3+^ doping conditions under the excitation of 980 nm laser.

UC luminescence spectroscopy was used to investigate the UC properties of the GdSr_2_AlO_5_: Yb, Er under 980 nm laser excitation. It can been seen from Fig. 5 that two UC emissions lied in 527 nm and 549 nm, which were assigned to ^2^H_11/2_ → ^4^I_15/2_ (Er^3+^) and ^4^S_3/2_ → ^4^I_15/2_ (Er^3+^) transitions, respectively.^26^ Meanwhile, the red UC emission line centered at 665 nm is observed, which comes from the transition of ^4^F_9/2_ → ^4^I_15/2_ (Er^3+^). As the Yb^3+^ doping concentration increased from 0 to 10%, the UC emission first rapidly increased and then decreased, when the Yb^3+^ doping concentration reach 6% we get the best emission.

Generally speaking, the absorption can be improved by increasing the doping concentration of the lanthanide ions in the material. But non-radiative multi-phonon relaxation can occur, and the process of cross-relaxation severely limits the range of useful dopant concentrations. Non-radiative multi-phonon relaxation rate between energy levels is another important factor that dictates the population of intermediate and emitting levels and subsequently determines the efficiency of the upconversion process. Therefore, when Yb^3+^ was co-doped into the lattice in high concentrations, the upconversion luminescence intensity decreased. It can been find that an elevated amount of Yb^3+^ content is likely to enhance the luminescence efficiency of UC as the Yb^3+^ ion has a sufficient absorption cross-section matched to common 980 nm laser excitation. Thereby, successive energy transfer processes from Yb^3+^ to Er^3+^ play an important role in promoting the Er^3+^ ion on the ground state to the excited state.^27^

Additional, we added the upconversion luminescence spectra of Yb and Er doped samples in order to prove the existence of the energy transfer between Yb and Er. From Fig. S2† we can find that the intensity for the upconversion luminescence of Yb, Er co-doped sample is much higher than the singly doped samples. This can be the evidence of the energy transfer between Yb and Er.

**Fig. 6:**
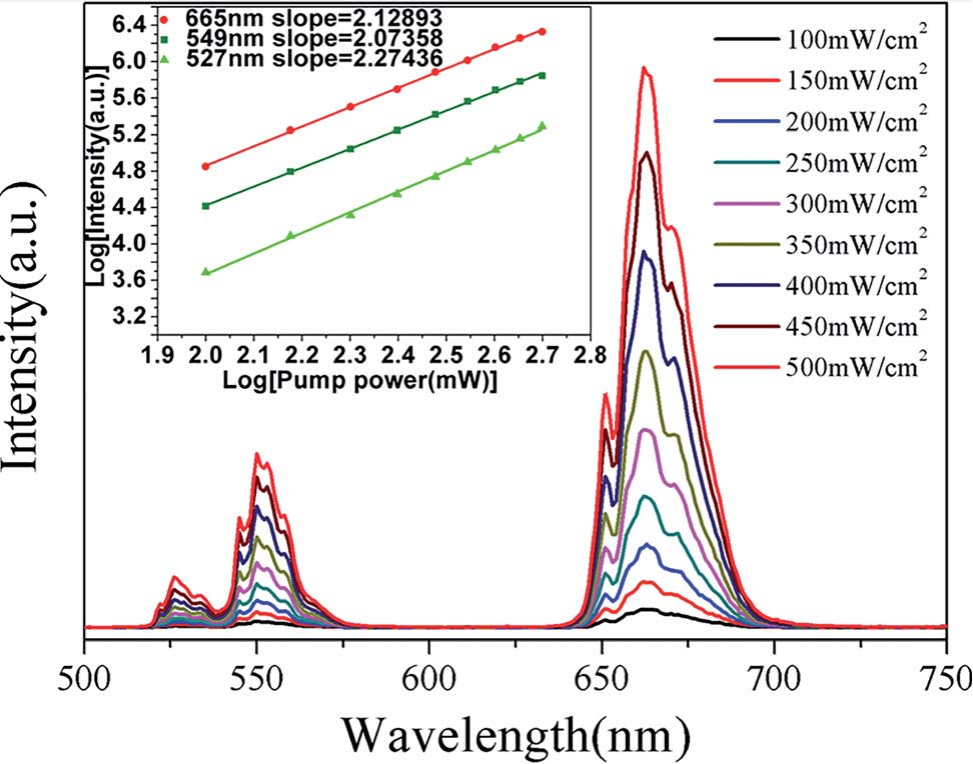
UC spectra of GdSr_2_AlO_5_:6%Yb^3+^/1%Er^3+^ and (inset)Ln–Ln plot of emission intensity of different excited states *versus* excitation power of GdSr_2_AlO_5_:6%Yb^3+^/1%Er^3+^.

These changes of UC luminescence intensity also means the successful incorporation of Yb^3+^ and Er^3+^ into the GdSr_2_AlO_5_ host. To get a better understanding of the UC luminescence mechanism, the power-dependent UC luminescence properties were studied. In any unsaturated UC process, it is generally considered that the visible emission intensity (*I*_up_) increases regularly according to the pumping power (*P*) (seen Fig. 6). From the UC luminescence spectra of the prepared Yb^3+^/Er^3+^ co-doped GdSr_2_AlO_5_ under the excitation of a 980 nm diode laser at different pumping powers (100–500 mW cm^−2^), we can easily find the intensity continued to increase as the pumping power increased.

The relationship between the intensity of UC luminescence *I*_up_ and the pump power *P* can be written as:^28–30^

1 *I*_up_ ∝ *P*^*N*^

where *N* is the order of multiphoton transitions, the number of infrared quanta absorbed per photon emission. From the inset in Fig. 5, we can find that the values of *N* for sample GdSr_2_AlO_5_:6%Yb^3+^/1%Er^3+^ are 2.27, 2.07 and 2.13 at 527 nm, 549 nm and 665 nm emissions, indicating 2 photons are needed to realize the transitions of ^2^H_11/2_ → ^4^I_15/2_ (527 nm), ^4^S_3/2_ → ^4^I_15/2_ (549 nm) and ^4^F_9/2_ → ^4^I_15/2_ (665 nm). Yb^3+^ has only two energy levels which match well with specific energy levels of Er^3+^ and it has much bigger absorption cross-section than Er^3+^ when excited with the 980 nm laser.

**Fig. 7:**
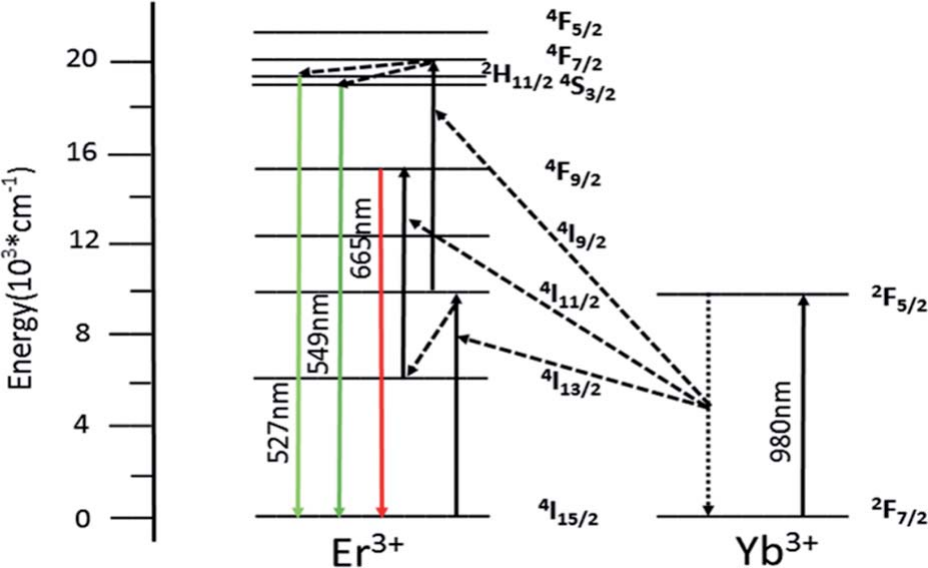
Implified energy level diagram of Yb^3+^ and Er^3+^ and the possible UC mechanism of GdSr_2_AlO_5_:6%Yb^3+^/1%Er^3+^.

Fig. 7 shows simplified energy level chart of the Yb^3+^, Er^3+^ as well as the proposed UC mechanisms to produce the multicolor radiation. Since the Yb ions concentration are much higher than Er ions, the most probable UC process is *via* energy transfer from the Yb ions to Er ions. When excited with laser light at 980 nm, the Yb ions are excited from the ^2^F_7/2_ level to the ^2^F_5/2_ level, and then transfer the energies to the nearby Er ions. In Yb^3+^/Er^3+^ co-doped GdSr_2_AlO_5_, firstly the Er^3+^ is excited from the ^4^I_15/2_ ground state to the ^4^I_11/2_ level and subsequently to the ^4^F_7/2_ level, and the nonradiative relaxations then populate the states of ^2^H_11/2_ and ^4^S_3/2_. Er^3+^ in ^4^I_13/2_ level are promoted to ^4^F_9/2_ level by the same energy transfer. Finally, the green emissions at 527 nm and 549 nm are generated by the ^2^H_11/2_ → ^4^I_15/2_ and ^4^S_3/2_ → ^4^I_15/2_ transitions of the Er^3+^, and the red emission centered at 665 nm is due to the ^4^F_9/2_ → ^4^I_15/2_ Er^3+^ transition of the Er^3+^.

### 3.3. Temperature sensing properties

**Fig. 8:**
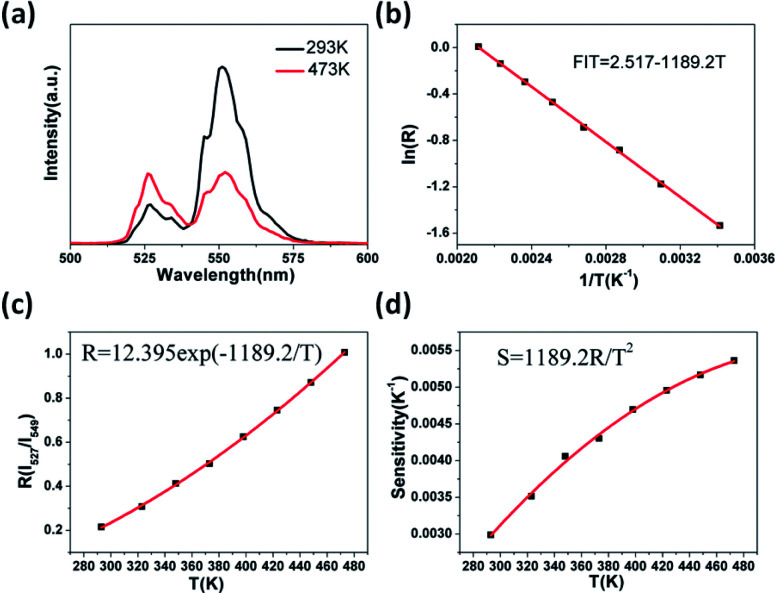
UC-based temperature sensing behavior of the GdSr_2_AlO_5_, (a) green UC emission spectra measured at 293 K and 473 K, (b) monolog plot of *R* (using 527 and 549 nm) as a function of inverse absolute temperature, (c) *R* relative to the absolute temperature, (d) the sensitivity as a function of the absolute temperature.

Fig. 8a shows the green UC emission spectra of Er^3+^/Yb^3+^ co-doped GdSr_2_AlO_5_ in the wavelength range of 500–600 nm at the measured temperatures of 293 K and 473 K. When the temperature increases, we can find the positions of the two green UC emission bands did not change. However, the *R*(*I*_527_/*I*_549_)of the two emissions bands varies at different temperature. When the temperature is 293 K, the emission intensity of the ^2^H_11/2_ → ^4^I_15/2_ band (527 nm) is lower than ^4^S_3/2_ → ^4^I_15/2_ (549 nm) but inverts at a higher temperature (473 K). The relative populations of the two thermally coupled levels, ^2^H_11/2_ and ^4^S_3/2_, follows a Boltzmann law, which leads to the variations in the intensities of the ^2^H_11/2_ → ^4^I_15/2_ and ^4^S_3/2_ → ^4^I_15/2_ transitions of the Er^3+^ ions at an raising temperature.

The emission intensity ratio *R* of the two thermally coupled Er^3+^ energy levels can be defined as:^31–33^

2 *R* = *I*_527_/*I*_549_ = *C* exp(−Δ*E*/*kT*)

where *I*_527_ and *I*_549_ are the integrated intensities from the ^2^H_11/2_ → ^4^I_15/2_ and ^4^S_3/2_ → ^4^I_15/2_ transitions of Er^3+^, respectively, Δ*E* is the energy separation between the ^2^H_11/2_ and ^4^S_3/2_ levels, *k* is the Boltzmann constant and *T* is the temperature (in K). Fig. 8b shows the change of the *R* for the emission bands centered at 527 nm and 549 nm as a function of the inverse absolute temperature on a monolog scale. The linear behavior of the curve shows the applicability of the material for temperature sensing applications.^34^ The ln *R* as a function of 1/*T* is perfectly fitted to a linear equation with the slope of −1189.2 and use the eqn (2) the *C* can be calculated. Thus, the expression of *R* could be determined as *R* = 12.395 exp(−1189.2/*T*) (see Fig. 8c). Furthermore, understanding the rate at which the measured temperature-sensitive parameter varies for a certain change in temperature is significance for temperature sensing applications. Hence the sensor sensitivity can be written as:

3 Sensitivity (S) = d*R*/d*T* = *R*(−Δ*E*/*kT*^2^)

Here the symbols have their usual meanings. The sensor sensitivity was calculated by this equation and it was plot ted in Fig. 8d. Impressively, the sensitivity increases gradually as the temperature rise from 293 K to 473 K, and reached the peak at about 0.0054 K^−1^at 473 K. The UC emission intensity is quite stable up to the measured temperature at 473 K, which suggests it may be suitable at higher temperatures as well. However as a lack of available devices the research at higher temperature could not be furthered done. Therefore, this study reveals that the GdSr_2_AlO_5_ host is a good material for UC-based temperature sensors.

**Fig. 9:**
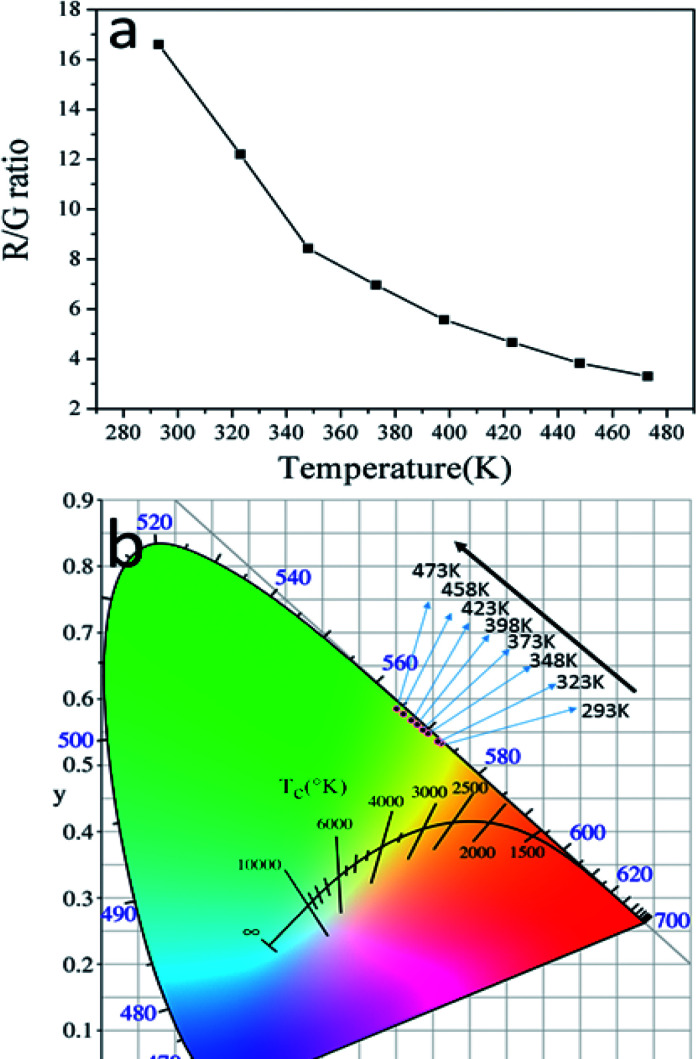
(a) The dependence of R/G ratio on temperature and (b) CIE coordinates GdSr_2_AlO_5_:6%Yb^3+^/1%Er^3+^ micro-particles in the temperature range of 293–473 K.

Besides the dependence of the *R*(*I*_red_/*I*_green_)ratio on temperature, the effect of temperature on the ratio of red and green emissions (R/G) were also observed. Fig. 9a shows the relationship between temperature and the R/G ratio. Evidently, the R/G ratio tends to decreases with the increase of the temperature. So the emission color of the sample could be changed by controlling the temperature. In order to insight the relationship between emission color of the sample and temperature more clearly, the CIE 1931 coordinates of the prepared GdSr_2_AlO_5_:6%Yb^3+^,1%Er^3+^ micro-particles in the range of temperatures 293–473 K were calculated from the recorded UC luminescence spectra and the corresponding results are shown in the CIE 1931 coordinates diagram in Fig. 9b. It can be seen that the UC emission color can be changed from yellow to green as the rise of temperature, this means a decrease tendency of the emission intensity ratio of red and green emissions can been observed with the increase of temperature. This is the same as the result shows in Fig. 9a.

It is known to all that the nonradiative transition rate of RE^3+^ ions for multiphonon relaxation can be written by the following equation:^35,36^

4 *W*_NR_ = *W*_NR_(0)exp−(Δ*E*/*kT*)

where *W*_NR_(0) is the nonradiative relaxation rate at 0 K, Δ*E* is the energy separation between the ^2^H_11/2_ and the ^4^S_3/2_ levels (700 cm^−1^), *k* is Boltzmann's constant, and *T* is the absolute temperature. And then, we can conclude that that the nonradiative transition rate will increase as the temperature increases. In Yb^3+^/Er^3+^ co-doped systems, actually, the emission intensities from all the transitions would decreases due to the multiphonon relaxation processes when the temperature increases. This means a significant decline of population on the ^4^F_9/2_ level due to the successively nonradiative processes of ^4^F_9/2_ → ^4^I_9/2_ → ^4^I_11/2_ → ^4^I_13/2_ → ^4^I_15/2_ are dominant at high temperature.

Thereby, both the green and red emission intensities will decrease greatly at higher temperature in normal situation. However, owing to the ^2^H_11/2_ and ^4^S_3/2_ levels are thermally coupled, much more electrons will be excited to the ^2^H_11/2_ level from the ^4^S_3/2_ level at high temperature. The occurrence of this phenomenon would block the de-excited process of the ^4^S_3/2_ level extremely *via* the nonradiative transition of ^2^H_11/2_, ^4^S_3/2_ → ^4^F_9/2_. Thence, the decrease of the total emission intensity of green color would be limited, this lead to the greatly decrease of the ratio of red and green emissions when the temperature arises. The temperature-dependent characteristic of the as-prepared GdSr_2_AlO_5_:Yb^3+^, Er^3+^ micro-particles means that this host could be also adopted as safety sign in high temperature environment as well as optical thermograph.

## 4. Conclusions

Micro-sized GdSr_2_AlO_5_:Yb^3+^/Er^3+^ particles with different doping concentrations of Yb^3+^ are designed and prepared by a high-temperature solid state reaction method. The UC luminescence properties of Yb^3+^/Er^3+^ co-doped GdSr_2_AlO_5_ upon 980 nm excitation have been systematically investigated. The UC emission spectra of GdSr_2_AlO_5_: Yb^3+^/Er^3+^ mainly contain two parts, the green emission peaks at 527 nm and 549 nm assigned to the (^2^H_11/2_, ^4^S_3/2_) → ^4^I_15/2_ transitions of the Er^3+^, and the red emission peak centered at 665 nm attributed to the Er^3+ 4^F_9/2_ → ^4^I_15/2_ transition, as a result, visible emission can be observed by the naked eye under 980 nm laser excitation. The intensity of the emission can be controlled by varying the Yb^3+^ concentrations. As a function of temperature ranging from 293 K to 473 K the green UC emission was tested and it has a sensitivity of 0.0054 K^−1^ at 473 K. Besides the UC emission color can be adjusted from the yellow to green, which could be regarded as another virtue of temperature-dependent UC emission color. In conclusion, the UC luminescence property and temperature dependence of GdSr_2_AlO_5_: Yb^3+^/Er^3+^ are reported for the first time and these characteristics indicate that the oxide host material GdSr_2_AlO_5_ has potential applications in UC-based optical temperature sensors as well as high temperature safety signal.

## Conflicts of interest

There are no conflicts to declare.

## Supplementary Material

RA-008-C7RA13759A-s001
