# Supplementary material for: An upconversion luminescence and temperature sensor based on Yb3+/Er3+ co-doped GdSr2AlO5
Source: RSC Adv. 2018 Mar 5;8(17):9512–8. doi: 10.1039/c7ra13759a (PMC9078722; doi:10.1039/c7ra13759a)

Electronic Supplementary Material (ESI) for RSC Advances.
This journal is © The Royal Society of Chemistry 2018

Upconversion luminescence and temperature sensor based on Yb^{3+}/Er^{3+} co-doped $GdSr_2AlO_5$

Gangyi Zhang[ab], Qinping Qiang[ab],Shanshan Du[ab] and Yuhua Wang [ab*]

[a]Department of Materials Science, School of Physical Science and Technology, Lanzhou University, Lanzhou, 730000, PR China and Key [b]Laboratory for Special Function Materials and Structural Design of the Ministry of Education, Lanzhou University, Lanzhou 730000, China.
[*]Corresponding author: Yuhua Wang
Email address: wyh@lzu.edu.cn; tel.: +86 931 8912772; fax: +86 931 8913554.

Figure S1 SEM image of $GdSr_2AlO_5$ host

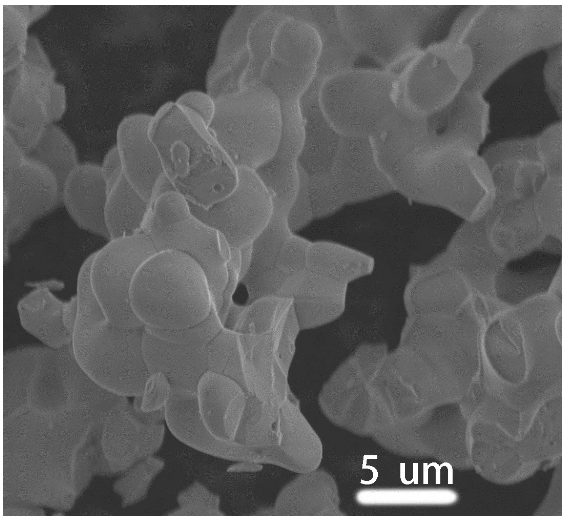

Figure S2 UC spectra of $GdSr_2AlO_5$:Yb^{3+},Er^{3+} crystals with different Yb^{3+} and Er^{3+} doping conditions under the excitation of 980 nm laser.

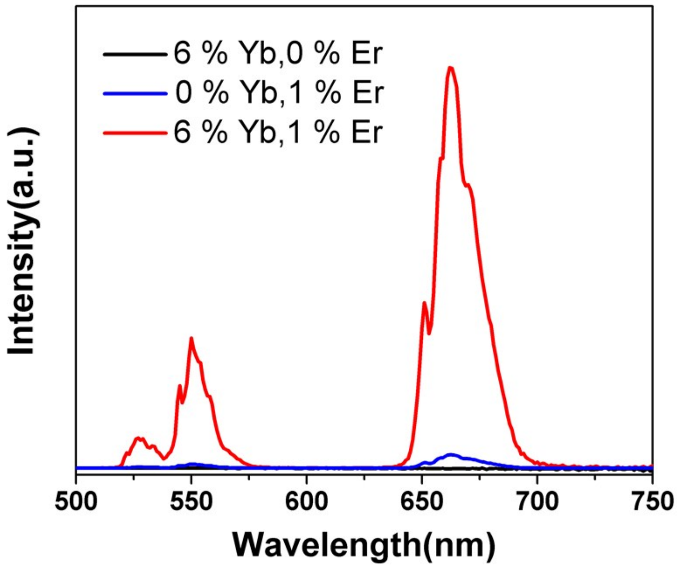

Supplement: RA-008-C7RA13759A-s001 [file RA-008-C7RA13759A-s001.pdf]
